# Neurological Manifestations in Parry–Romberg Syndrome: 2 Case Reports

**DOI:** 10.1097/MD.0000000000001147

**Published:** 2015-07-17

**Authors:** Justine Vix, Stéphane Mathis, Mathieu Lacoste, Rémy Guillevin, Jean-Philippe Neau

**Affiliations:** From Department of Neurology, CHU Poitiers, University of Poitiers, Poitiers (JV, SM, JPN); Cabinet of Neurology, Niort (ML); and Department of Radiology, CHU Poitiers, University of Poitiers, Poitiers, France (RG).

## Abstract

Parry–Romberg syndrome (PRS) is a variant of morphea usually characterized by a slowly progressive course. Clinical and radiological involvement of the central nervous system may be observed in PRS.

We describe 2 patients with PRS and neurological symptoms (one with trigeminal neuralgia associated with deafness, and the second with hemifacial pain associated with migraine without aura) in conjunction with abnormal cerebral MRI including white matter T2 hyperintensities and enhancement with gadolinium. Despite the absence of specific immunosuppressive treatments, both patients have presented stable imaging during follow-up without any clinical neurologic progression. We have performed a large review of the medical literature on patients with PRS and neurological involvement (total of 129 patients)

Central nervous system involvement is frequent among PRS patients and is inconsistently associated with clinical abnormalities. These various neurological manifestations include seizures, headaches, movement disorders, neuropsychological symptoms, and focal symptoms. Cerebral MRI may reveal frequent abnormalities, which can be bilateral or more often homolateral to the skin lesions, localized or so widespread so as to involve the whole hemisphere: T2 hyperintensities, mostly in the subcortical white matter, gadolinium enhancement, brain atrophy, and calcifications. These radiological lesions do not usually progress over time. Steroids or immunosuppressive treatments are controversial since it remains unclear to what extent they are beneficial and there is often no neurological progression.

## INTRODUCTION

Parry–Romberg syndrome (PRS), which is also known as “progressive facial hemiatrophy,” was first described by Caleb Parry in 1825 and by Moritz Romberg in 1846.^1^ As a localized scleroderma, it is usually characterized by a benign prognosis and the absence of significant internal organ lesions. However, evidence of clinical and radiological involvement of the central nervous system (CNS) has increased over the years.^1,2^ Herein, we have described 2 cases of PRS in which neurological involvement is demonstrated, with a large-scale review of the medical literature.

## METHODS

We have observed 2 patients with neurological symptoms and PRS. A review of the literature of neurological complications of PRS has also been performed by searching in *Pubmed*, *Google Scholar*, and *Sciencedirect* (from 1995 to 2015) with the terms: [neurological complications AND Parry–Romberg syndrome] OR [neurology AND Parry–Romberg syndrome] OR [pain AND Parry–Romberg syndrome] OR [MRI AND Parry–Romberg syndrome] OR [brain AND Parry–Romberg syndrome] OR [neurological complications AND en coup de sabre] OR [neurology AND en coup de sabre] OR [pain AND en coup de sabre] OR [MRI AND en coup de sabre] OR [brain AND en coup de sabre].

Among all the case reports and series of cases found, we have selected only those with enough clinical (sex of the patient, age at onset of disease, age at onset of neurological symptoms, and description of neurological symptoms), radiological (brain CT scan description and/or brain MRI description), and if possible biological (cerebrospinal fluid: cells count, protein level and oligoclonal bands) data.

## CASE REPORTS

### Patient 1

A 55-year-old man had come to our outpatient clinic for 10 years as he was suffering from right trigeminal neuropathic pain with facial hypoesthesia and paresthesia, associated with right deafness. The pain was located in the right V2 distribution (lower part of the right nostril, cheekbone, cheek, and superior hemilip). He most often compared his pain to an electrical discharge that lasted from a few seconds to several minutes and more rarely described it as a continuous dull and achy, or latent pain. He rated its intensity between 2 and 7 on a 10-point verbal rating scale. The pain was triggered by chewing, tooth brushing of the right upper gum, shaving of the right upper hemilip, and more rarely by shampooing of the hair. He experienced several shooting pains every day during a few days to 2 weeks several times a year. There was no nausea, photophobia, phonophobia, or osmophobia associated with the pain, nor was it exacerbated by movement. On clinical examination, he also presented with slightly decreased pupillary reactivity in the right eye (ophthalmological examination was normal), as well as right hypoacousia and a trigeminal loss of sensation to light touch on the right part of the upper lip.

**FIGURE 1:**
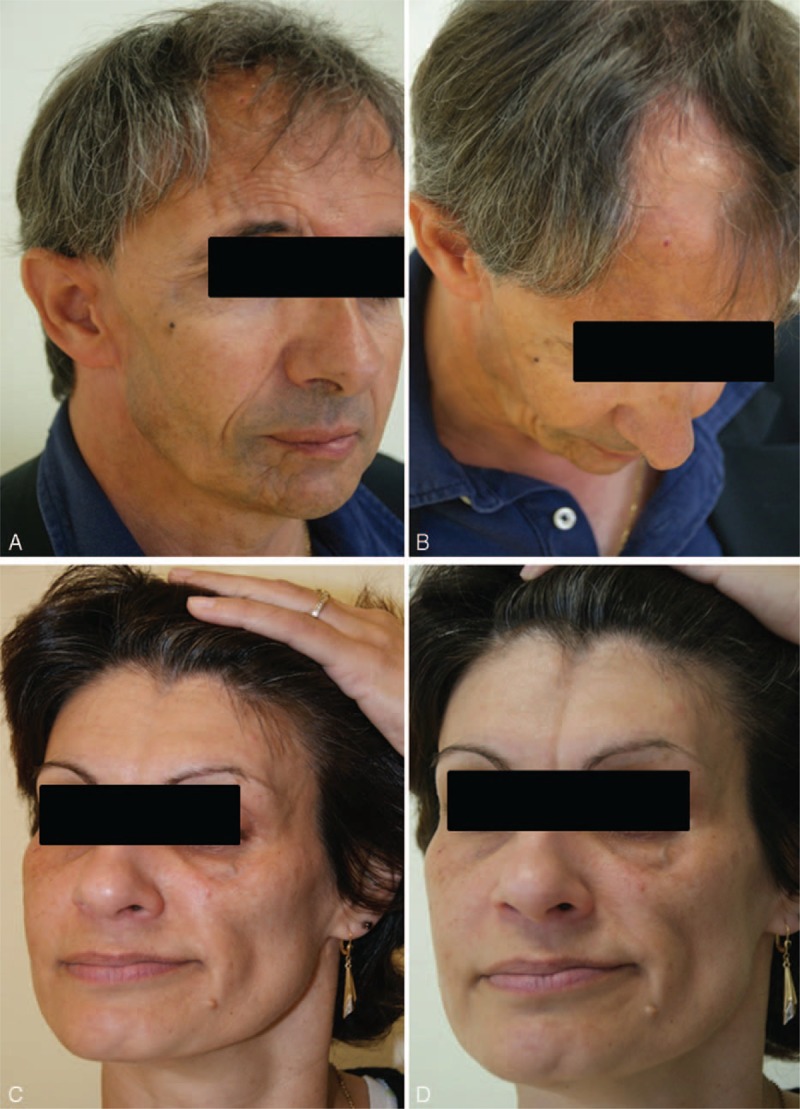
Case 1 (A and B): right facial atrophy with frontal linear scleroderma *en coup de sabre* (A) and an area of alopecia (B). Case 2 (C and D): left facial atrophy with ptosis (C) and *en coup de sabre* forehead (D).

He had no specific medical past except when he was 10, a right hemifacial atrophy that started out with a typical lipoatrophic lesion on the forehead and, after a delay of several years, progressed to overt progressive hemifacial atrophy (for which he twice underwent surgery). No further progression of his hemifacial atrophy was noted after the age of 20. At the time of our examination, there was an atrophy of the soft tissue affecting the entire right part of his face including the chin, upper and lower lips, tongue, cheek, zygomatic area, and forehead associated with a frontal linear scleroderma “en coup de sabre” and an area of alopecia (Figure 1A and B).

**FIGURE 2:**
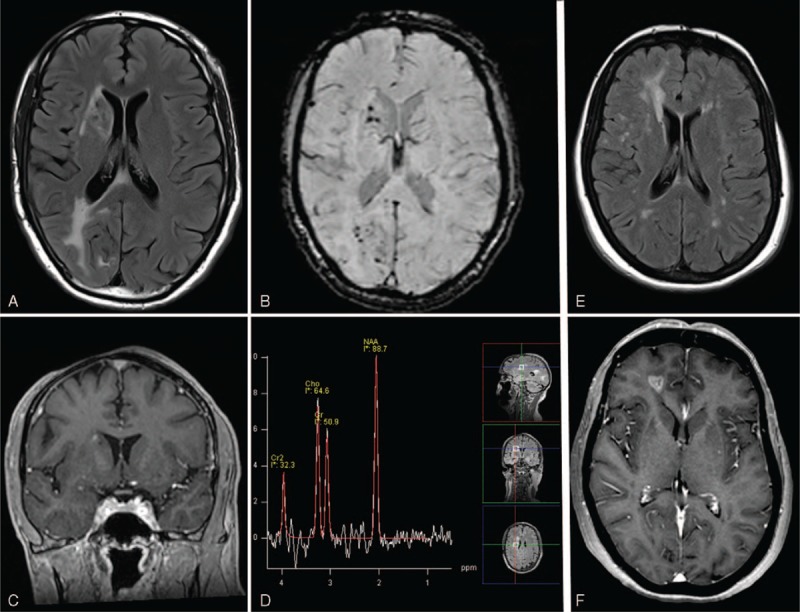
Case 1 (A–D): MRI axial T2-FLAIR (A): hyperintensities of the right hemisphere affecting posterior part of the corpus callosum, occipital white matter, anterior limb of the internal capsule, and caudate nucleus. MRI axial susceptibility weighted imaging (B): numerous right microhemorrhages of the caudate nucleus and occipital area. MRI with coronal T1 postcontrast image shows a small gadolinium enhancement of the right several caudate nucleus (C). MR proton spectroscopy: markedly decreased NAA resonance associated with a slightly increased choline resonance without free lipids or lactate resonance (D). Case 2 (E and F): MRI axial T2-FLAIR (E): large hyperintensity of the right hemisphere affecting frontal white matter and multiple bilateral small lesions of the white matter. MRI with axial T1 postcontrast image with heterogeneous gadolinium enhancement of the right frontal white matter (F).

Initial magnetic resonance imaging (June 2011) showed an extended area of T2-FLAIR hyperintensities of the right hemisphere located mostly amid the posterior occipital white matter, but also in the posterior part of the corpus callosum, the caudate nucleus and the anterior limb of the internal capsule (Figure 2A). Gradient echo-images and susceptibility weighted imaging sequences showed numerous microbleeds disseminated throughout the deep part of the right hemisphere (caudate nucleus, pallidum) and the occipital white matter (Figure 2B). There was also a slight patchy enhancement following gadolinium injection of the caudate nucleus (Figure 2C). Notwithstanding a right cerebellar atrophy, there was no abnormality of the left hemisphere or left posterior fossa. There was no abnormality of retro-orbital fat, and angio-MRI was normal. A second MRI performed 3 years later (November 2014) disclosed no modification. On the other hand, MR proton spectroscopy demonstrated markedly decreased NAA resonance and slightly increased choline resonance without free lipids or lactate resonance (Figure 2D). The different metabolic abnormalities were consistent with sequelar lesions.

He was successfully treated for his trigeminal pain with carbamazepine and aspirin.

### Patient 2

This 44-year-old woman suffered for several years from left facial tension-type pain with acute exacerbations located on her cheek and radiating toward her left upper lip and gum. She also occasionally suffered from acute migraine attacks without aura, lasting several hours, essentially located on her left side. Nonsteroidal anti-inflammatory drugs were effective. Neurological and ophthalmological examinations were normal.

In her past medical history, she had been diagnosed 10 years earlier with progressive left hemifacial atrophy (Figure 1C) without any pain but presenting an “en coup de sabre” lesion on her forehead (Figure 1D).

Because of her left hemifacial pain and atrophy, a cerebral MRI was performed and disclosed mostly contralateral abnormalities with right multiple and diffused T2-FLAIR hyperintensity lesions, primarily located in the frontal lobe (Figure 2E). Some of these lesions presented gadolinium enhancement (Figure 2F). Left small hyperintensities of the left white matter were likewise discovered. There was no abnormality of retro-orbital fat. No infratentorial lesion, microhemorrhages on gradient echo-images, cerebral atrophy or, vascular malformation on angio-MRI was found.

While lumbar puncture was normal (white cell count and protein level), oligoclonal bands were present.

During follow-up, cerebral MRI performed at 6 and 12 months showed no progression of the cerebral lesions.

## DISCUSSION

Parry–Romberg syndrome (PRS) is a rare acquired neurocutaneous disorder characterized by progressive hemifacial atrophy. It commonly affects the dermatomes of 1 or multiple branches of the trigeminal nerve with atrophy of the skin and underlying structures (soft tissues, muscles, and bones), and can also touch the eye (enophthalmos, uveitis, retinal vasculitis, glaucoma, central retinal artery occlusion, heterochromic iridocyclitis, restrictive strabismus, Coats disease, papillitis, optic atrophy, and neuroretinitis), the pupil (Horner syndrome, pupillary abnormalities), and hair (band-like alopecia).^3^ It is typically restricted to one half of the face, but occasionally involves the arm, trunk, and leg,^3^ and is sometimes bilateral.^4^ Its incidence ranges from 0.3 to 2.5 cases per 100,000 population per year,^5^ most likely less than 3/100,000,^6^ and is pronouncedly more common in women, who represent more than 3 of 4 of the patients.^2,4^ PRS usually begins in the first decade of life,^2,3^ although late-onset cases have also been described,^4,7^ and slowly progresses over 2 to 20 years before stabilizing. The final degree of deformity may depend on the duration of the disease. Finally, PRS is sporadic, although some rare familial cases have been reported.^8^

We have analyzed a total of 129 patients (in 82 medical articles) with PRS and neurological involvement: our 2 patients, 69 single case reports,^1,9–75^ 7 reports of 2 cases,^76–82^ 2 reports of 3 cases,^83,84^ 2 reports of 4 cases,^85,86^ 1 report of 7 cases,^87^ and 1 report of 23 cases.^2^ There were 81 women (62.8%) and 48 men (37.2%). The average age at onset of PRS was 13.2 (1–69) years in the whole population, 10.2 (1–39) years in male, and 15.5 (1–69) years in female. The mean age at onset of the neurological symptoms was 20.9 (1.5–73) years in the whole population, 15.2 (2–45) years in male, and 24.5 (1.5–73) years in female. The neurological symptoms appeared after the onset of PRS in most of cases (69.7%), with an average delay of 11.6 (0.5–44) years. Rarely, neurological symptoms preceded the onset of PRS (13.2%), with an average delay of 5.9 (1.5–9) years. The onset of neurological symptoms and PRS was the same (delay <6 months between the 2 occurrences) in 13.9% of the patients.

The main neurological signs and symptoms of Parry-Romberg syndrome are reported in (Table 1) [and the main radiological abnormalities in (Table 2)], indicating that CNS involvement represents the most frequent systemic manifestation, thereby justifying its classification as a neurocutaneous syndrome.^12^

**TABLE 1:**
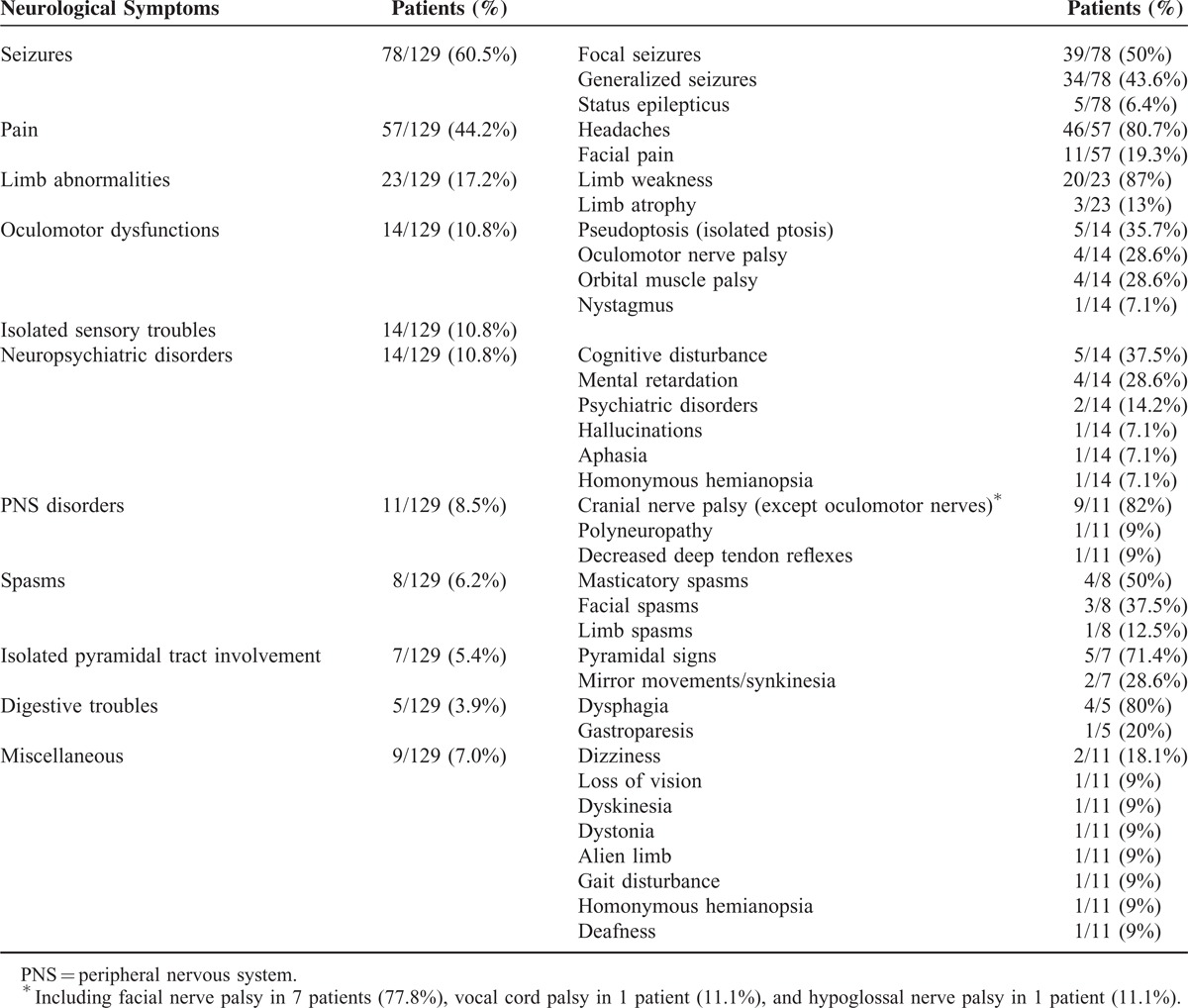
Main Neurological Symptoms in Parry–Romberg Syndrome

**TABLE 2:**
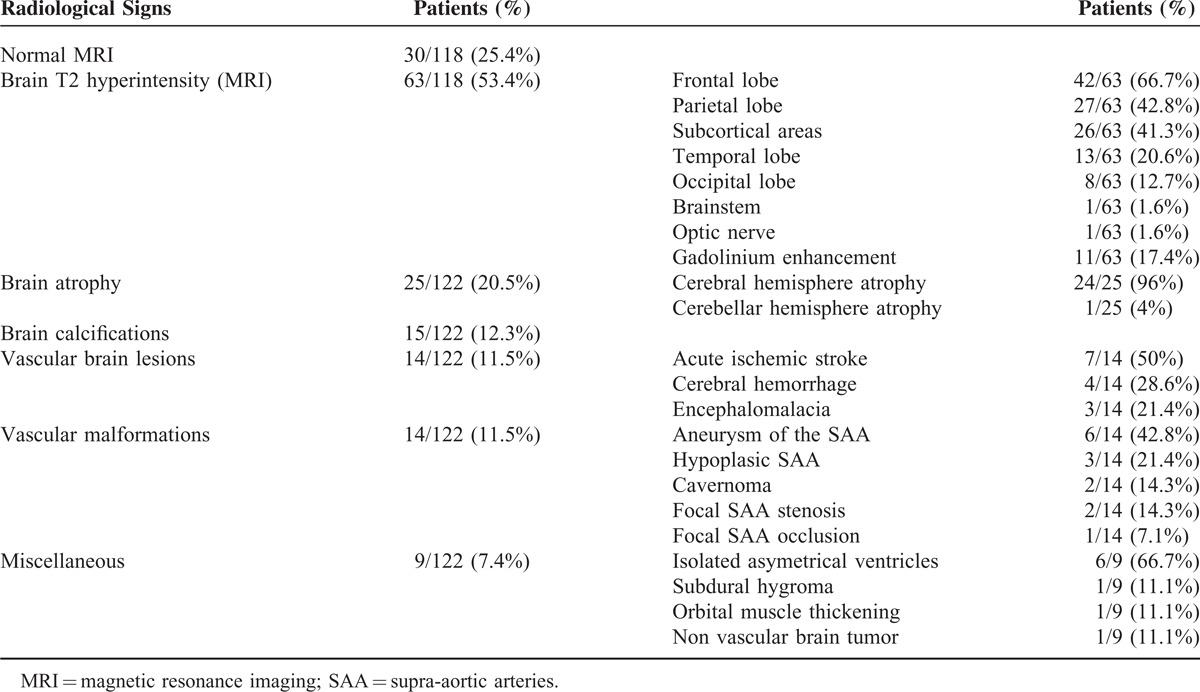
Main Radiological Findings in Parry–Romberg Syndrome

In our review of the literature, epilepsy is the most common cerebral manifestation (60.5%), and was known to be present at onset of CNS involvement in 58% of the cases and in 73% over the course of PRS neurological manifestations.^1^ Epilepsy consists of focal seizures in 50% of the patients and generalized seizures in 43.6% of the patients. In 33% of patients, these seizures are refractory to medication.^1,79,87^ Some patients have presented a clinical picture of cerebral hemisphere atrophy, intractable focal seizures, with brain biopsy findings of perivascular inflammation^45^ and microglial nodules indistinguishable from Rasmussen encephalitis.^61,88^ In focal seizures, the side of brain involvement and the side of the cutaneous lesion are usually the same, but cases with discordance on the side of involvement have also been reported, albeit more rarely.^31,45^ In most cases, electroencephalogram is abnormal (48% of the patients), with frontal and temporal discharges or slowing and generalized abnormalities, with or without cerebral lesions on neuroradiological examinations. An electroencephalogram study (EEG) has been performed in only 75 patients: 36 patients (48%) have an abnormal EEG, mostly linked to seizures (89.3%); 10.1% of the patients have abnormal EEG (slow wave activity) without acute seizure or medical past history of seizure.

Headaches are also frequent (pain and headache represent, respectively, 44.2% and 35.6% of the neurological symptoms), but any relationship between migraine among young patients (primarily women) and PRS is rather questionable, although it seems more frequent in PRS than in the general population (affecting 52% of 205 patients with PRS surveyed using the Internet).^4^ In addition, migraine^50^ can lead to hemiplegia,^89,90^ at times associated with reversible angiographic changes.^73^ Severe migrainosus state, with frontoparietal white matter lesions on MRI, has likewise been described.^41^ In addition, facial pain^4,91^ and trigeminal neuralgia^23,77,88,91^ have been commonly reported: we have found facial pain in 11 patients (8.5% of the patients).

Cerebral vascular malformations have also been mentioned as occurring in PRS (11.5%). Cerebral aneurysms remain rare,^2,11,14,35^ however, their actual incidence remains unknown, since not all of the patients examined have undergone head CT scan, brain MRI, or cerebral angiogram. Their location is variable, as they may affect the internal carotid artery^11^ or the intracranial vessels,^35,50,60,92^ and they are exceptionally revealed by a subarachnoid hemorrhage.^35^ Nevertheless, since no pathological studies have been carried out, the pathogenesis of these aneurysms has yet to be fully understood.^11^ Two different hypotheses have nonetheless been postulated; one involves a failure of neural crest migration that may explain vascular abnormalities and aneurysm formation;^50,60,92^ the other posits localized vasculitis leading to fragility of the arterial wall,^35^ and is apparently supported by proliferative interstitial neurovasculitis on histological examinations of facial specimens from patients with PRS.^35^ Spontaneous carotid-jugular fistula and carotid dissection,^35,93^ hypoplastic arteries associated with homolateral phthisis bulbi,^51^ and brain cavernomas^80^ complete the wide-ranging gamut of these cerebrovascular malformations. Since all of them are located in the affected side, their coincidence seems highly unlikely. Even though PRS may involve the cerebral or cervical vessels, symptomatic ischemic strokes are rare, only found in 7 of patients (5.7%).^20,32,65^ Motor deficit may be sudden or insidiously progressive due to focal stenosis in the M2 portion of the middle cerebral artery, leading to cerebral infarct in the watershed region with secondary extension to deep white matter accompanied by scattered hemorrhages.^32^

Other neurological CNS manifestations have been more episodically described: limb (contralateral, ipsilateral) and trunk atrophy,^4^ cerebral microhemorrhages,^94^ cerebellar hemorrhage,^2^ movement disorders (including paroxysmal kinesigenic dyskinesia,^46^ torticollis,^95^ mandibular cramps,^77^ dystonia,^71^ and facial^2^ or masticatory^66^ spasms), facial nerve palsy^26^ (that could also be due to local atrophy),^1^ dysphonia (vocal cord palsy),^52^ sympathetic hyperactivity,^23^ bilateral pyramidal tract involvement,^96^ mirror movements,^69^ brain tumor,^13^ alien limb,^18^ cognitive disturbances, and progressive mental retardation due to cerebral atrophy (with or without focal seizures).^1,28,79^ Ocular motility disturbances may also be observed, due to restrictive myopathy (in part inflammatory)^97^ and oculomotor palsies.^98^ Occasionally, intraorbital fat atrophy has been reported by local imaging (echography or MRI), or histological analysis (when strabismus surgery).^75,99^

A lumbar puncture has been performed in 20 patients, and the CSF analysis was normal in all of them with a normal protein level, and an average cells count equal to 2.1 cells/mm^3^; however, a slight pleiocytosis (<11/mm^3^) may be observed.^48^ As in our second patient, oligoclonal bands and an elevated IgG index (often associated with enhancing brain lesions on MRI)^1^ may be found in 50% of the patients (without other biological abnormality).

Finally, a brain imaging has been performed in 122 patients (CT scan and/or MRI), and a MRI in 118 patients (Table 2). Among these 118 patients with neurological manifestations, brain MRI was normal in 30 patients (25.4%). In 75% of the patients with PRS and neurological manifestations, CT and most brain MRI revealed several abnormalities, the cerebral lesions at times being subtle or extended; they are bilateral in nearly two-thirds of the patients, strictly ipsilateral to the cutaneous involvement in one third, and more rarely contralateral to cutaneous findings.^2^ Bone deformity and outer diploe thinning in the frontoparietal region underlying the area of more severe skin lesion are usually observed on CT scan.^85^ On MRI T1 sequence (or CT scanner), cerebral atrophy may be observed (20.5% of the patients), paralleling that of the skin. Focal cortical atrophy, asymmetry of the lateral ventricle,^2^ blurring of the gray-white matter interface, and cortical thickening are common and predominate in the anterior region.^85^ Porencephalic cyst and encephalomalacia has likewise been described.^2^ Atrophy may possibly be more diffuse and widespread, involving the entire cerebral hemisphere^1,28^ or the cerebellum.^85^ T2 hyperintensities essentially affect frontal and subcortical white matter, and more rarely the corpus callosum, deep gray nuclei, brainstem, and optic nerves.^1^ As in our patients, enhancement on CT or MRI has been reported in some patients (in 17.4% of patients with brain hyperintensity) and is mostly associated with intrathecal antibody production in CSF. It can persist and even increase over a number of years despite immunosuppressive treatments.^1,4,20^ Finally, brain calcifications are also observed (12.3%) and may involve the basal ganglia, thalami, and dentate nuclei.^85^ While they are characteristically ipsilateral to the skin lesions, they may also be contralateral.^4,53,100^ The above-mentioned abnormalities can be present among asymptomatic patients and new lesions may be found on follow-up brain MRI demonstrating subclinical progression in some cases.^1^ MRA or cerebral angiograms may demonstrate vascular changes suggestive of vasculitis in some patients (11.5%),^1,28,73^ hypoplastic arteries,^28,42^ cerebral aneurysms,^2^ and other vascular malformations, which could constitute the late sequelae of a vasculitic process.

Other neurological explorations such as brain SPECT may be likely to detect abnormalities in PRS patients^28^ who have had normal brain MRI^101^ and show no clinical manifestations, thereby demonstrating that subclinical CNS involvement is present in most, and perhaps even all patients with craniofacial scleroderma.^1^

Skin and brain biopsy have disclosed a similar inflammatory process. Brain biopsy, which is rarely performed, has shown these inflammatory changes to exist in vessel walls and/or parenchyma; on the basis of clinical presentation and biopsy findings, it is sometimes diagnosed as Rasmussen encephalitis.^4,45,61,78,83^

Given the rarity of localized scleroderma, there are currently no standardized treatments for PRS and its underlying inflammatory lesions. In cases of localized scleroderma, methotrexate and corticosteroids are the treatment of choice, while other immunosuppressant agents such as cyclophosphamide, cyclosporine, D-penicillamine, rituximab,^45^ and hydroxychloroquine are used when more aggressive therapy is required.^3,4,40^ The extent to which these treatments are beneficial^4^ nevertheless remains unclear, as the majority of patients having undergone serial imaging have presented stable if occasionally controversial^1^ imaging findings.^2,102^

Although the exact pathophysiological mechanism of localized scleroderma and PRS is still unknown, an immune mediated process has been postulated, supported by inflammatory changes in the parenchyma and vessel walls in brain biopsies, a frequent oligoclonal response in the CSF, occasional coexistence with autoimmune disorders (vitiligo, thyroid problems, systemic sclerosis, inflammatory bowel disease, rheumatoid arthritis, ankylosing spondylitis, and lupus),^4^ and improvement or resolution of lesions following immunosuppression.

In conclusion, overt neurologic involvement appears to be not nearly as infrequent as previously believed since clinical symptoms have been reported by as many as half of the patients with craniofacial scleroderma. However, this percentage certainly represents an underestimation, and we deem it advisable to undertake a complete clinical neurological examination, EEG and MRI of all patients exhibiting hemifacial atrophy, even when there are no neurological signs. Long-term follow-up of PRS patients is required, given the evidence that neurologic abnormalities may occasionally develop at any time over the course of the disease. Further research into the efficacy of immunosuppressants would be strongly recommended.

## References

[1] Kister I Inglese M Laxer RM Neurologic manifestations of localized scleroderma: a case report and literature review. *Neurology* 2008; 71:1538–1545. 18981376 10.1212/01.wnl.0000334474.88923.e3

[2] Doolittle DA Lehman VT Schwartz KM CNS imaging findings associated with Parry–Romberg syndrome and en coup de sabre: correlation to dermatologic and neurologic abnormalities. *Neuroradiology* 2014; 57:21–34. 25304124 10.1007/s00234-014-1448-6

[3] El-Kehdy J Abbas O Rubeiz N A review of Parry–Romberg syndrome. *J Am Acad Dermatol* 2012; 67:769–784. 22405645 10.1016/j.jaad.2012.01.019

[4] Stone J Parry-Romberg syndrome: a global survey of 205 patients using the Internet. *Neurology* 2003; 61:674–676. 12963760 10.1212/wnl.61.5.674

[5] Bielsa Marsol I Update on the classification and treatment of localized scleroderma. *Actas Dermosifiliogr* 2013; 104:654–666. 23948159 10.1016/j.adengl.2012.10.012

[6] Peterson LS Nelson AM Su WP The epidemiology of morphea (localized scleroderma) in Olmsted County 1960-1993. *J Rheumatol* 1997; 24:73–80. 9002014

[7] Mendonca J Viana SL Freitas F Late-onset progressive facial hemiatrophy (Parry-Romberg syndrome). *J Postgrad Med* 2005; 51:135–136. 16006711

[8] Lewkonia RM Lowry RB Progressive hemifacial atrophy (Parry-Romberg syndrome) report with review of genetics and nosology. *Am J Med Genet* 1983; 14:385–390. 6601461 10.1002/ajmg.1320140220

[9] Aktekin B Oguz Y Aydin H Cortical silent period in a patient with focal epilepsy and Parry-Romberg syndrome. *Epilepsy Behav* 2005; 6:270–273. 15710317 10.1016/j.yebeh.2004.11.012

[10] de Paula RA Ribeiro BN Bahia PR Parry-Romberg syndrome: findings in advanced magnetic resonance imaging sequences—case report. *Radiol Bras* 2014; 47:186–188. 25741077 10.1590/0100-3984.2013.1699 PMC4337135

[11] Aoki T Tashiro Y Fujita K Parry-Romberg syndrome with a giant internal carotid artery aneurysm. *Surg Neurol* 2006; 65:170–173. 16427416 10.1016/j.surneu.2005.05.006

[12] Aynaci FM Sen Y Erdol H Parry-Romberg syndrome associated with Adie's pupil and radiologic findings. *Pediatr Neurol* 2001; 25:416–418. 11744320 10.1016/s0887-8994(01)00333-2

[13] Bergler-Czop B Lis-Swiety A Brzezinska-Wcislo L Scleroderma linearis: hemiatrophia faciei progressiva (Parry-Romberg syndrom) without any changes in CNS and linear scleroderma “en coup de sabre” with CNS tumor. *BMC Neurol* 2009; 9:39. 19635150 10.1186/1471-2377-9-39 PMC2723072

[14] Bosman T Van Bei Jnum J Van Walderveen MA Giant intracranial aneurysm in a ten-year-old boy with parry romberg syndrome. A case report and literature review. *Interv Neuroradiol* 2009; 15:165–173. 20465894 10.1177/159101990901500205 PMC3299017

[15] Budrewicz S Koszewicz M Koziorowska-Gawron E Parry-Romberg syndrome: clinical, electrophysiological and neuroimaging correlations. *Neurol Sci* 2012; 33:423–427. 21909746 10.1007/s10072-011-0756-4 PMC3313041

[16] Chak G Wang HZ Feldon SE Coup de sabre presenting with worsening diplopia and enophthalmos. *Ophthal Plast Reconstr Surg* 2011; 27:e97–e98. 10.1097/IOP.0b013e3181f29c86 21750423

[17] Chiang KL Chang KP Wong TT Linear scleroderma “en coup de sabre”: initial presentation as intractable partial seizures in a child. *Pediatr Neonatol* 2009; 50:294–298. 20025145 10.1016/S1875-9572(09)60081-4

[18] Chokar G Cerase A Gough A A case of Parry-Romberg syndrome and alien hand. *J Neurol Sci* 2014; 341:153–157. 24793509 10.1016/j.jns.2014.04.004

[19] Chung MH Sum J Morrell MJ Intracerebral involvement in scleroderma en coup de sabre: report of a case with neuropathologic findings. *Ann Neurol* 1995; 37:679–681. 7755364 10.1002/ana.410370519

[20] Cory RC Clayman DA Faillace WJ Clinical and radiologic findings in progressive facial hemiatrophy (Parry-Romberg syndrome). *AJNR Am J Neuroradiol* 1997; 18:751–757. 9127045 PMC8338508

[21] Dalla Costa G Colombo B Dalla Libera D Parry Romberg syndrome associated with chronic facial pain. *J Clin Neurosci* 2013; 20:1320–1322. 23528409 10.1016/j.jocn.2012.08.020

[22] DeFelipe J Segura T Arellano JI Neuropathological findings in a patient with epilepsy and the Parry-Romberg syndrome. *Epilepsia* 2001; 42:1198–1203. 11580770 10.1046/j.1528-1157.2001.45800.x

[23] Drummond PD Hassard S Finch PM Trigeminal neuralgia, migraine and sympathetic hyperactivity in a patient with Parry-Romberg syndrome. *Cephalalgia* 2006; 26:1146–1149. 16919067 10.1111/j.1468-2982.2006.01161.x

[24] Duyff RF Vos J A “scar” and epilepsy: coup de sabre. *J Neurol Neurosurg Psychiatry* 1998; 65:568. 9771788 10.1136/jnnp.65.4.568 PMC2170264

[25] Ehmann D Riyaz R Greve M Central retinal artery occlusion in a child with Parry-Romberg syndrome. *Can J Ophthalmol* 2014; 49:e9–e10. 24513383 10.1016/j.jcjo.2013.09.012

[26] Gambichler T Kreuter A Hoffmann K Bilateral linear scleroderma “en coup de sabre” associated with facial atrophy and neurological complications. *BMC Dermatol* 2001; 1:9. 11741509 10.1186/1471-5945-1-9 PMC61032

[27] Goldberg-Stern H deGrauw T Passo M Parry-Romberg syndrome: follow-up imaging during suppressive therapy. *Neuroradiology* 1997; 39:873–876. 9457714 10.1007/s002340050525

[28] Grosso S Fioravanti A Biasi G Linear scleroderma associated with progressive brain atrophy. *Brain Dev* 2003; 25:57–61. 12536035 10.1016/s0387-7604(02)00147-x

[29] Higashi Y Kanekura T Fukumaru K Scleroderma en coup de sabre with central nervous system involvement. *J Dermatol* 2000; 27:486–488. 10935351

[30] Holl-Wieden A Klink T Klink J Linear scleroderma ’en coup de sabre’ associated with cerebral and ocular vasculitis. *Scand J Rheumatol* 2006; 35:402–404. 17062443 10.1080/03009740600556126

[31] Kakisaka Y So NK Jones SE Intractable focal epilepsy contralateral to the side of facial atrophy in Parry-Romberg syndrome. *Neurol Sci* 2012; 33:165–168. 21647628 10.1007/s10072-011-0643-z

[32] Kanzato N Matsuzaki T Komine Y Localized scleroderma associated with progressing ischemic stroke. *J Neurol Sci* 1999; 163:86–89. 10223417 10.1016/s0022-510x(98)00267-6

[33] Karim A Laghmari M Ibrahimy W Neurorétinite, hémiatrophie faciale progressive de Parry-Romberg et sclérodermie localisée. A propos d’un cas et revue de la littérature. *J Fr Ophtalmol* 2005; 28:866–870. 16249769 10.1016/s0181-5512(05)81008-3

[34] Kasapcopur O Ozkan HC Tuysuz B Linear scleroderma en coup de sabre and brain calcification: is there a pathogenic relationship? *J Rheumatol* 2003; 30:2724–2725. 14719224

[35] Kuechler D Kaliaperumal C Hassan A Aneurysmal subarachnoid haemorrhage in Parry-Rhomberg syndrome. *BMJ Case Rep* 2011; pii:bcr1020114920. 10.1136/bcr.10.2011.4920 PMC3229419 22674607

[36] Panda AK Gopinath G Singh S Parry-Romberg syndrome with hemimasticatory spasm in pregnancy: a dystonia mimic. *J Neurosci Rural Pract* 2014; 5:184–186. 24966565 10.4103/0976-3147.131675 PMC4064192

[37] Longo D Paonessa A Specchio N Parry-Romberg syndrome and Rasmussen encephalitis: possible association. Clinical and neuroimaging features. *J Neuroimaging* 2011; 21:188–193. 19555404 10.1111/j.1552-6569.2009.00398.x

[38] Madruga Dias J Costa MM Pereira da Silva JA Parry-Romberg syndrome in an 11-year-old female with neurological manifestations without brain imaging abnormalities. *Joint Bone Spine* 2012; 79:419–421. 22366144 10.1016/j.jbspin.2012.01.005

[39] Malandrini A Dotti MT Federico A Selective ipsilateral neuromuscular involvement in a case of facial and somatic hemiatrophy. *Muscle Nerve* 1997; 20:890–892. 9179164 10.1002/(sici)1097-4598(199707)20:7<890::aid-mus16>3.0.co;2-w

[40] Maletic J Tsirka V Ioannides P Parry-Romberg syndrome associated with localized scleroderma. *Case Rep Neurol* 2010; 2:57–62. 20671858 10.1159/000314927 PMC2905582

[41] Menascu S Padeh S Hoffman C Parry-Romberg syndrome presenting as status migrainosus. *Pediatr Neurol* 2009; 40:321–323. 19302950 10.1016/j.pediatrneurol.2008.11.007

[42] Miedziak AI Stefanyszyn M Flanagan J Parry-Romberg syndrome associated with intracranial vascular malformations. *Arch Ophthalmol* 1998; 116:1235–1237. 9747688 10.1001/archopht.116.9.1235

[43] Miller MT Spencer MA Progressive hemifacial atrophy. A natural history study. *Trans Am Ophthalmol Soc* 1995; 93:203–215. 8719679 PMC1312058

[44] Moon WJ Kim HJ Roh HG Diffusion tensor imaging and fiber tractography in Parry-Romberg syndrome. *AJNR Am J Neuroradiol* 2008; 29:714–715. 18202229 10.3174/ajnr.A0967 PMC7978183

[45] Moseley BD Burrus TM Mason TG Neurological picture. Contralateral cutaneous and MRI findings in a patient with Parry-Romberg syndrome. *J Neurol Neurosurg Psychiatry* 2010; 81:1400–1401. 20802222 10.1136/jnnp.2009.202044

[46] Mrabet Khiari H Masmoudi S Mrabet A Association syndrome de Parry-Romberg et dyskinésie paroxystique kinésigénique. *Rev Neurol (Paris)* 2009; 165:489–492. 18930510 10.1016/j.neurol.2008.08.003

[47] Obermoser G Pfausler BE Linder DM Scleroderma en coup de sabre with central nervous system and ophthalmologic involvement: treatment of ocular symptoms with interferon gamma. *J Am Acad Dermatol* 2003; 49:543–546. 12963929 10.1067/s0190-9622(03)00901-0

[48] Paprocka J Jamroz E Adamek D Difficulties in differentiation of Parry-Romberg syndrome, unilateral facial sclerodermia, and Rasmussen syndrome. *Childs Nerv Syst* 2006; 22:409–415. 16247619 10.1007/s00381-005-1262-x

[49] Park DH Kim IT Patient with Parry-Romberg syndrome complicated by Coats’ syndrome. *Jpn J Ophthalmol* 2008; 52:520–522. 19089584 10.1007/s10384-008-0597-8

[50] Pichiecchio A Uggetti C Grazia Egitto M Parry-Romberg syndrome with migraine and intracranial aneurysm. *Neurology* 2002; 59:606–608. 12196658 10.1212/wnl.59.4.606

[51] Qureshi UA Wani NA Altaf U Parry-Romberg syndrome associated with unusual intracranial vascular malformations and Phthisis bulbi. *J Neurol Sci* 2010; 291:107–109. 20144465 10.1016/j.jns.2010.01.003

[52] Rafai MA Boulaajaj FZ El Moutawakil B Syndrome de Parry-Romberg avec dysphonie. *Rev Neurol (Paris)* 2007; 163:1246–1248. 18355475 10.1016/S0035-3787(07)78412-6

[53] Rosario C Garelick D Greenberg G Plaque morphea with neurological involvement: an extraordinary uncommon presentation. *Clin Rheumatol* 2013; 34:597–601. 24352753 10.1007/s10067-013-2458-1

[54] Rudolph G Haritoglou C Kalpadakis P Hemifacial atrophy (Parry-Romberg syndrome, #141300) with papillitis, retinal alterations, and restriction of motility. *J AAPOS* 2002; 6:126–129. 11997811 10.1067/mpa.2002.122520

[55] Sahin MT Baris S Karaman A Parry-Romberg syndrome: a possible association with borreliosis. *J Eur Acad Dermatol Venereol* 2004; 18:204–207. 15009307 10.1111/j.1468-3083.2004.00862.x

[56] Salpetrio D Merlino M Bruglia S Linear scleroderma en coup de sabre associated with facial atrophy in a patient seropositive for Borrelia burgdorferi: a true case of molecular mimicry? *Pediatr Allergy Immunol* 2004; 15:570–572. 15610374 10.1111/j.1399-3038.2004.00189.x

[57] Sandhu K Handa S Subdural hygroma in a patient with Parry-Romberg syndrome. *Pediatr Dermatol* 2004; 21:48–50. 14871326 10.1111/j.0736-8046.2004.21109.x

[58] Sartori S Martini G Calderone M Severe epilepsy preceding by four months the onset of scleroderma en coup de sabre. *Clin Exp Rheumatol* 2009; 27:64–67. 19796565

[59] Sathornsumetee S Schanberg L Rabinovich E Parry-Romberg syndrome with fatal brain stem involvement. *J Pediatr* 2005; 146:429–431. 15756237 10.1016/j.jpeds.2004.10.026

[60] Schievink WI Mellinger JF Atkinson JL Progressive intracranial aneurysmal disease in a child with progressive hemifacial atrophy (Parry-Romberg disease): case report. *Neurosurgery* 1996; 38:1237–1241. 8727157 10.1097/00006123-199606000-00038

[61] Shah JR Juhasz C Kupsky WJ Rasmussen encephalitis associated with Parry-Romberg syndrome. *Neurology* 2003; 61:395–397. 12913207 10.1212/wnl.61.3.395

[62] Slimani S Hounas F Ladjouze-Rezig A Multiple linear sclerodermas with a diffuse Parry-Romberg syndrome. *Joint Bone Spine* 2009; 76:114–116. 18993106 10.1016/j.jbspin.2008.07.009

[63] Straube A Padovan CS Seelos K Parry-Romberg syndrom und Rasmussen-Syndrom: nur zufallige Ahnlichkeiten? *Nervenarzt* 2001; 72:641–646. 11519207 10.1007/s001150170066

[64] Strenge H Cordes P Sticherling M Hemifacial atrophy: a neurocutaneous disorder with coup de sabre deformity, telangiectatic naevus, aneurysmatic malformation of the internal carotid artery and crossed hemiatrophy. *J Neurol* 1996; 243:658–660. 8892068 10.1007/BF00878663

[65] Tomizawa Y Tanaka R Sekiguchi K Cerebral infarction in a case of Parry-Romberg syndrome. *J Stroke Cerebrovasc Dis* 2014; 23:393–394. 23664460 10.1016/j.jstrokecerebrovasdis.2013.02.015

[66] Unterberger I Trinka E Engelhardt K Linear scleroderma “en coup de sabre” coexisting with plaque-morphea: neuroradiological manifestation and response to corticosteroids. *J Neurol Neurosurg Psychiatry* 2003; 74:661–664. 12700315 10.1136/jnnp.74.5.661 PMC1738439

[67] Verhelst HE Beele H Joos R Hippocampal atrophy and developmental regression as first sign of linear scleroderma “en coup de sabre”. *Eur J Paediatr Neurol* 2008; 12:508–511. 18207439 10.1016/j.ejpn.2007.12.001

[68] Verma R Ram H Gupta M A case of extensive left-sided facial atrophy of Romberg. *Natl J Maxillofac Surg* 2013; 4:77–80. 24163557 10.4103/0975-5950.117881 PMC3800390

[69] Verma R Dixit PK Lalla R Mirror movements in progressive hemifacial atrophy. *Ann Indian Acad Neurol* 2015; 18:246–248. 26019431 10.4103/0972-2327.150606 PMC4445209

[70] Viana M Glastonbury CM Sprenger T Trigeminal neuropathic pain in a patient with progressive facial hemiatrophy (Parry-Romberg syndrome). *Arch Neurol* 2011; 68:938–943. 21747035 10.1001/archneurol.2011.126

[71] Walker RH Fink JK Morphea and Parry-Romberg syndrome associated with a mixed movement disorder. *Parkinsonism Relat Disord* 2013; 19:1169–1170. 23968650 10.1016/j.parkreldis.2013.07.025 PMC4045497

[72] Waters MF Bhidayasiri R Shields WD Favorable longitudinal outcome in a patient with Parry-Romberg syndrome. *Acta Neurol Scand* 2005; 112:192–193. 16097963 10.1111/j.1600-0404.2005.00452.x

[73] Woolfenden AR Tong DC Norbash AM Progressive facial hemiatrophy: abnormality of intracranial vasculature. *Neurology* 1998; 50:1915–1917. 9633763 10.1212/wnl.50.6.1915

[74] Yano T Sawaishi Y Toyono M Progressive facial hemiatrophy after epileptic seizures. *Pediatr Neurol* 2000; 23:164–166. 11020643 10.1016/s0887-8994(00)00168-5

[75] Zubcov-Iwantscheff AA Thomke F Goebel HH Eye movement involvement in Parry-Romberg syndrome: a clinicopathologic case report. *Strabismus* 2008; 16:119–121. 18788061 10.1080/09273970802240874

[76] Anderson PJ Molony D Haan E Familial Parry-Romberg disease. *Int J Pediatr Otorhinolaryngol* 2005; 69:705–708. 15850693 10.1016/j.ijporl.2004.12.004

[77] Brito JC Holanda MM Holanda G Hemiatrofia facial progressiva (doenca de Parry-Romberg). Relato de dois casos associados a trigeminalgia e caimbras. *Arq Neuropsiquiatr* 1997; 55:472–477. 9629367 10.1590/s0004-282x1997000300020

[78] Carreno M Donaire A Barcelo MI Parry Romberg syndrome and linear scleroderma in coup de sabre mimicking Rasmussen encephalitis. *Neurology* 2007; 68:1308–1310. 17438222 10.1212/01.wnl.0000259523.09001.7a

[79] Chbicheb M Gelot A Rivier F Parry-Romberg's syndrome and epilepsy. *Rev Neurol (Paris)* 2005; 161:92–97. 15678008 10.1016/s0035-3787(05)84980-x

[80] Fain ET Mannion M Pope E Brain cavernomas associated with en coup de sabre linear scleroderma: two case reports. *Pediatr Rheumatol Online J* 2011; 9:18. 21801349 10.1186/1546-0096-9-18 PMC3162908

[81] Menni S Marzano AV Passoni E Neurologic abnormalities in two patients with facial hemiatrophy and sclerosis coexisting with morphea. *Pediatr Dermatol* 1997; 14:113–116. 9144696 10.1111/j.1525-1470.1997.tb00216.x

[82] Seifert F Bien CG Schellinger PD Parry-Romberg syndrome with chronic focal encephalitis: two cases. *Clin Neurol Neurosurg* 2011; 113:170–172. 21093978 10.1016/j.clineuro.2010.10.009

[83] Holland KE Steffes B Nocton JJ Linear scleroderma en coup de sabre with associated neurologic abnormalities. *Pediatrics* 2006; 117:e132–e136. 16326691 10.1542/peds.2005-0470

[84] Ruiz-Sandoval JL Romero-Vargas S Gutierrez-Aceves GA Esclerodermia lineal en coup de sabre. Manifestaciones neurologicas, imagenes y revision. *Rev Neurol* 2005; 41:534–537. 16254860

[85] Appenzeller S Montenegro MA Dertkigil SS Neuroimaging findings in scleroderma en coup de sabre. *Neurology* 2004; 62:1585–1589. 15136686 10.1212/01.wnl.0000124518.25087.18

[86] Dupont S Catala M Hasboun D Progressive facial hemiatrophy and epilepsy: a common underlying dysgenetic mechanism. *Neurology* 1997; 48:1013–1018. 9109892 10.1212/wnl.48.4.1013

[87] Sommer A Gambichler T Bacharach-Buhles M Clinical and serological characteristics of progressive facial hemiatrophy: a case series of 12 patients. *J Am Acad Dermatol* 2006; 54:227–233. 16443052 10.1016/j.jaad.2005.10.020

[88] Marzano AV Menni S Parodi A Localized scleroderma in adults and children. Clinical and laboratory investigations on 239 cases. *Eur J Dermatol* 2003; 13:171–176. 12695134

[89] Sagild JC Alving J Hemiplegic migraine and progressive hemifacial atrophy. *Ann Neurol* 1985; 17:620. 4026238 10.1002/ana.410170621

[90] Ong B Chong PN Yeo PP Progressive hemifacial atrophy: a report of 2 cases. *Singapore Med J* 1990; 31:497–499. 2259953

[91] Kumar AA Kumar RA Shantha GP Progressive hemi facial atrophy: Parry Romberg syndrome presenting as severe facial pain in a young man: a case report. *Cases J* 2009; 2:6776. 19829858 10.4076/1757-1626-2-6776 PMC2740286

[92] Catala M Progressive intracranial aneurysmal disease in a child with progressive hemifacial atrophy (Parry-Romberg disease): case report. *Neurosurgery* 1998; 42:1195–1196. 9588570 10.1097/00006123-199805000-00161

[93] Schievink WI Piepgras DG Nichols DA Spontaneous carotid-jugular fistula and carotid dissection in a patient with multiple intracranial arachnoid cysts and hemifacial atrophy: a generalized connective tissue disorder? Case report. *J Neurosurg* 1995; 83:546–549. 7666235 10.3171/jns.1995.83.3.0546

[94] Blitstein MK Tung GA MRI of cerebral microhemorrhages. *AJR Am J Roentgenol* 2007; 189:720–725. 17715122 10.2214/AJR.07.2249

[95] Kee C Hwang JM Parry-Romberg syndrome presenting with recurrent exotropia and torticollis. *J Pediatr Ophthalmol Strabismus* 2008; 45:368–370. 19043950 10.3928/01913913-20081101-10

[96] Miao J Liu R Lin H Severe bilateral pyramidal tract involvement in a patient with Parry-Romberg syndrome. *Am J Med Sci* 2009; 337:212–214. 19174689 10.1097/MAJ.0b013e31818226f9

[97] Khan AO Restrictive strabismus in Parry-Romberg syndrome. *J Pediatr Ophthalmol Strabismus* 2007; 44:51–52. 17274338 10.3928/01913913-20070101-09

[98] Prescott CR Hasbani MJ Levada AJ Ocular motor dysfunction in Parry-Romberg syndrome: four cases. *J Pediatr Ophthalmol Strabismus* 2011; 48:e63–e66. 22149072 10.3928/01913913-20111129-02

[99] Bandello F Rosa N Ghisolfi F New findings in the Parry-Romberg syndrome: a case report. *Eur J Ophthalmol* 2002; 12:556–558. 12510729 10.1177/112067210201200620

[100] Fry JA Alvarellos A Fink CW Intracranial findings in progressive facial hemiatrophy. *J Rheumatol* 1992; 19:956–958. 1404134

[101] Blaszczyk M Krolicki L Krasu M Progressive facial hemiatrophy: central nervous system involvement and relationship with scleroderma en coup de sabre. *J Rheumatol* 2003; 30:1997–2004. 12966605

[102] Careta MF Leite Cda C Cresta F Prospective study to evaluate the clinical and radiological outcome of patients with scleroderma of the face. *Autoimmun Rev* 2013; 12:1064–1069. 23791631 10.1016/j.autrev.2013.05.005

